# APC/C-regulated CPT1C promotes tumor progression by upregulating the energy supply and accelerating the G1/S transition

**DOI:** 10.1186/s12964-024-01657-z

**Published:** 2024-05-23

**Authors:** Huihui Zhao, Xinxin Cheng, Liping Yan, Fang Mi, Wenqing Wang, Yuying Hu, Xingyang Liu, Yuyan Fan, Qingjie Min, Yan Wang, Weimin Zhang, Qingnan Wu, Qimin Zhan

**Affiliations:** 1 Key Laboratory of Carcinogenesis and Translational Research (Ministry of Education/Beijing), Laboratory of Molecular Oncology, Peking University Cancer Hospital & Institute, Beijing, 100142 China; 2 State Key Laboratory of Molecular Oncology, Beijing Key Laboratory of Carcinogenesis and Translational Research, Laboratory of Molecular Oncology, Peking University Cancer Hospital & Institute, Beijing, 100142 China; 3 Peking University International Cancer Institute, Beijing, 100142 China; 4 Institute of Cytology and Genetics, School of Basic Medical Sciences, Hengyang Medical School, University of South China, Hengyang, Hunan 421001 China; 5 Soochow University Cancer Institute, Suzhou, 215000 China; 6 State Key Laboratory of Molecular Oncology, National Cancer center/National Clinical Research Center for Cancer/Cancer Hospital, Chinese Academy of Medical Sciences and Peking Union Medical College, Beijing, 100021 China

**Keywords:** Cell cycle, Tumor progression, Fatty acid utilization, APC/C, CPT1C

## Abstract

**Background:**

In addition to functioning as a precise monitoring mechanism in cell cycle, the anaphase-promoting complex/cyclosome (APC/C) is reported to be involved in regulating multiple metabolic processes by facilitating the ubiquitin-mediated degradation of key enzymes. Fatty acid oxidation is a metabolic pathway utilized by tumor cells that is crucial for malignant progression; however, its association with APC/C remains to be explored.

**Methods:**

Cell cycle synchronization, immunoblotting, and propidium iodide staining were performed to investigate the carnitine palmitoyltransferase 1 C (CPT1C) expression manner. Proximity ligation assay and co-immunoprecipitation were performed to detect interactions between CPT1C and APC/C. Flow cytometry, 3-(4,5-dimethylthiazol-2-yl)-5-(3-carboxymethoxyphenyl)-2-(4-sulfophenyl)-2 H-tetrazolium, inner salt (MTS) assays, cell-scratch assays, and transwell assays and xenograft transplantation assays were performed to investigate the role of CPT1C in tumor progression in vitro and in vivo. Immunohistochemistry was performed on tumor tissue microarray to evaluate the expression levels of CPT1C and explore its potential clinical value.

**Results:**

We identified CPT1C as a novel APC/C substrate. CPT1C protein levels exhibited cell cycle-dependent fluctuations, peaking at the G1/S boundary. Elevated CPT1C accelerated the G1/S transition, facilitating tumor cell proliferation in vitro and in vivo. Furthermore, CPT1C enhanced fatty acid utilization, upregulated ATP levels, and decreased reactive oxygen species levels, thereby favoring cell survival in a harsh metabolic environment. Clinically, high CPT1C expression correlated with poor survival in patients with esophageal squamous cell carcinoma.

**Conclusions:**

Overall, our results revealed a novel interplay between fatty acid utilization and cell cycle machinery in tumor cells. Additionally, CPT1C promoted tumor cell proliferation and survival by augmenting cellular ATP levels and preserving redox homeostasis, particularly under metabolic stress. Therefore, CPT1C could be an independent prognostic indicator in esophageal squamous cell carcinoma.

**Supplementary Information:**

The online version contains supplementary material available at 10.1186/s12964-024-01657-z.

## Background

The cell cycle is a sequential and orchestrated process that is under the precise control of specific surveillance mechanisms. Those surveillance mechanisms, involving cell cycle checkpoints and anaphase-promoting complex/cyclosome (APC/C), enable progression to the next phase of the cell cycle only after a given phase is completed [1]. There is an internal mechanism in the late G1 phase, known as the restriction point, that determines whether cells advance through the G1/S boundary or exit cell cycle progression [2]. Entry into S phase typically requires a proper cell size, intact genomic DNA, and a functional extracellular matrix [3–6]. Moreover, sufficient material foundation and sufficient energy supply are crucial for a successful G1/S transition [2, 7].

APC/C is a conserved multi-subunit E3 ubiquitin ligase that plays critical roles in controlling cell cycle progression by binding to two coactivators, namely cell division cycle protein 20 (CDC20) and CDC20 homolog 1 (CDH1) [8]. The APC/C-CDC20 complex is activated at the onset of mitosis to trigger chromosome segregation and mitotic exit, whereas the APC/C-CDH1 complex is activated during late mitosis and the early G1 phase to ensure a stable G1 phase after mitotic exit, in turn helping establish optimal conditions before DNA replication in S phase [9]. Usually, APC/C binds to and degrades its substrates by recognizing particular amino acid sequences of the substrate proteins, such as a destruction box (D-box, RXXLXXXXE/D/N, where X can be any amino acid) and a lysine, glutamic acid, and asparagine (KEN) box [10]. Beyond imposing the correct temporal order on cell cycle progression, the APC/C appears to provide a linking mechanism that orchestrates cell cycle progression and metabolic activities. Previous findings have shown that APC/C regulates glycolysis and glutaminolysis in HeLa cells via the ubiquitin-mediated degradation of glutaminase 1 (GLS1) and 6-phosphofructo-2-kinase/fructose-2,6-biphosphatase 3 (PFKFB3) [11]. Moreover, APC/C maintains the balance of the dNTP pool by regulating thymidine kinase 1 and thymidylate kinase [12]. Previously, we demonstrated that APC/C coordinated the tricarboxylic acid (TCA) cycle at late G1 phase in esophageal squamous cell carcinoma (ESCC) [13]. Considering these noteworthy discoveries, the potential involvement of APC/C in regulating other metabolic pathways merits further study.

Increasing evidence shows that metabolic reprogramming contributes to cancer development and progression by increasing energy production, accelerating cell proliferation, enhancing metastasis, inducing immune escape, and conferring therapy resistance [14, 15]. In highly proliferating tumor cells, an expanded metabolic repertoire provides the flexibility needed to thrive in harsh tumor microenvironment such as those involving hypoxia and nutrient scarcity [16–18]. Although glucose is the major growth source, cancer cells take advantage of other metabolic products such as fatty acids and lactate to fuel cell growth, especially under nutrient- and oxygen-depleted environmental conditions [19, 20]. Recent data indicated that ovarian cancer cells used fatty acids for proliferation and metastasis [21]. Moreover, increased fatty acid utilization promoted stemness maintenance and drug resistance in breast cancer and lymphoma [22–24].

Fatty acid oxidation (FAO) is the main pathway for fatty acid catabolism in cells, supporting cellular energetics and redox homeostasis by ATP and NADPH, respectively [25]. Carnitine palmitoyltransferase 1 (CPT1), the rate-limiting enzyme in FAO, catalyzes the conversion of the long-chain molecule, acyl-coenzyme A (CoA), to acyl-carnitine, which facilitates fatty acid shuttling into the mitochondrial matrix [26]. There are three members in CPT1 family, namely CPT1A, CPT1B, and CPT1C. Since the roles of CPT1A and CPT1B in promoting cancer progression by upregulating FAO have been widely studied [24, 27, 28], attention has shifted to the recently discovered CPT1C. A growing number of studies are being conducted to clarify the function of CPT1C in tumors, including promoting proliferation and metastasis, and alleviating cellular lipotoxicity and senescence progression [29–31]. However, the association between CPT1C and the cell cycle remains unknown.

In this study, we conducted a comprehensive set of analyses and experiments to explore whether and how CPT1C interferes with cell cycle progression, as well as how it can overcome nutrient-depleted environmental conditions. We discovered that CPT1C, unlike other CPT1 family members, contains both a canonical KEN box and a D-box motif. Notably, only CPT1C protein levels fluctuated in a cell cycle-dependent manner, accumulating specifically at the G1/S boundary. We subsequently confirmed that CPT1C is a *bona fide* substrate of the APC/C-CDH1 and APC/C-CDC20 complexes. Furthermore, CPT1C accelerated the G1/S transition and facilitated tumor proliferation in vitro and in vivo. CPT1C promoted cell survival under metabolic stress by increasing fatty acid uptake and intracellular ATP production and maintaining redox homeostasis. Clinically, esophageal squamous cell carcinoma (ESCC) patients with high expression levels of CPT1C represented significantly poor prognosis. Therefore, our study revealed a novel crosstalk between fatty acid utilization and cell cycle machinery in tumor cells.

## Materials and methods

### Cell culture and treatment

HeLa cells were cultured in Dulbecco’s modified Eagle’s medium (Lonza, Switzerland) added with 10% fetal bovine serum (FBS; Gibco, USA) and 1% penicillin/streptomycin (Gibco). All human esophageal squamous cell carcinoma (ESCC) cell lines were cultured in RPMI-1640 medium (Lonza) added with 10% FBS and 1% penicillin/streptomycin (Gibco). All cell lines were cultured at 37 °C sterile incubator containing 5% CO_2_.

To induce G2/M phase arrest, cells were incubated with 200 ng/mL nocodazole (Sigma, Germany) for 18 h. To induce a double-thymidine block (DTB), cells were incubated with 2 mM thymidine (Sigma) for 14 h, fresh medium for the next 10 h, and then with 2 mM thymidine for another 14 h. We also used MG132 (20 µM, Sigma) and cycloheximide (100 µg/mL, MedChemExpress) in our cell-culture experiments.

### Plasmids and small-interfering RNA (siRNA) transfections and lentivirus infections

CPT1C-flag plasmid, which contains the full-length coding region of CPT1C fused with triple flag tags at the C-terminus, were purchased the from Mailgene Biosciences Co., Ltd. A lentivirus encoding CPT1C was constructed by Shanghai Jikai Gene Chemical Technology Co., Ltd. Cells were infected with the CPT1C-overexpressing lentivirus using polybrene (8 µg/mL) to enhance the infection efficiency. We used 2 µg/mL puromycin to select cells stably expressing CPT1C after infection. A Lipofectamine 2000 transfection kit (Invitrogen) was used to transfect plasmids and siRNAs into cells. CDC20-myc and CDH1-myc plasmids were previously constructed in our laboratory [32]. SiRNAs against CDH1 (siCDH1) and CDC20 (siCDC20) were provided from Guangzhou RiboBio Co., Ltd., and the target sequences are listed as follows: siCDH1-1: 5′-GCGTGAACTTCCACAGGAT-3′; siCDH1-2: 5′-GCTCCCAAGTGTGCAATCT-3′; siCDC20-1: 5′-GCAGAAACGGCTTCGAAAT-3′; and siCDC20-2: 5′-GGCGCTGTTTTGAGTTGGA-3′.

### Immunoblotting and immunoprecipitation

For immunoblotting, cell lysates were prepared using 1% NP-40 buffer added with a protease inhibitor cocktail (Roche, Germany). Denatured lysates were separated by SDS-PAGE and transferred onto PVDF membranes. After blocking the membranes with 5% BSA for 1 h at room temperature, they were incubated with the indicated primary antibodies at 4 °C overnight and then with horseradish peroxidase-conjugated secondary antibodies at room temperature for 1 h. The primary antibodies used are listed in Supplementary Table S1. Amersham Imager 600 (GE Healthcare, USA) was used to detect chemiluminescence signals.

For immunoprecipitation (IP) assays, cells were lysed in 1% NP-40 buffer added a protease inhibitor cocktail. The cell lysates were incubated with protein A/G-Sepharose beads (Thermo Fisher Scientific, USA) and the indicated primary antibodies or IgG at 4 °C, with shaking overnight. The beads were washed with NT-2 buffer. Immunoprecipitated samples were analyzed by immunoblotting.

### Cell cycle analysis

Cells were harvested via trypsin digestion, washed with pre-chilled phosphate-buffered saline (PBS), and then fixed in 75% pre-chilled ethanol at -20 °C overnight. The samples were washed thrice with PBS and resuspended in PI/RNase staining buffer (BD, 550,825). The samples were then incubated at room temperature in the dark for 30 min. Finally, the cells were filtered through 400 mesh sieves and analyzed using an Accuri C6 flow cytometer (BD Biosciences). An analysis of the percentage distribution of cell cycles was done using ModFit LT (Verity Software House, USA).

### Proximity-ligation assay (PLA)

The PLA was performed to detect protein-protein interactions in situ within 30 nm using the Proximity Ligation Assay Kit (Sigma-Aldrich, DUO92101) according to the manufacturer’s instructions. HeLa cells were seeded one day beforehand in glass-bottomed dishes. Following fixation in 4% paraformaldehyde for 15 min at room temperature, the cells were washed with PBS, blocked in Duolink blocking solution at 37 °C for 1 h, and then incubated with primary antibodies, CPT1C and CDC20, CPT1C and CDH1, diluted in Duolink Antibody Diluent at 4 °C overnight. After incubating the samples with PLA probes, ligases, and polymerases, the cells were washed and fluorescence images were captured with a confocal microscope (Leica ST2, Germany).

### 5-ethynyl-2′-deoxyuridine (EdU) assay

According to the manufacturer’s instructions, the EdU Kit (Beyotime, C0071s) was used to evaluate the proportion of cells in S phase. Cells were transfected for 48 h and incubated with 10 µM EdU for 2 h. After removing the medium, the samples were fixed for 15 min and permeabilized for 10 min at room temperature. The samples were washed with PBS, then incubated at room temperature for 30 min with click reaction solution. The samples were washed thrice and the number of EdU-positive cells was measured using a BD Accuri C6 flow cytometer.

### 3-(4,5-dimethylthiazol-2-yl)-5-(3-carboxymethoxyphenyl)-2-(4-sulfophenyl)-2 H-tetrazolium, inner salt (MTS) assay

Cell proliferation was detected via an MTS assay (Promega, Madison, WI, USA) according to the manufacturer’s instructions. 3 × 10^3^ cells per well were seeded in 96-well plates with transfected and control cells. At the indicated time points, 10 µL MTS reagent (15 mg/mL) was added into each well, and the cells were incubated for 1 h at 37 °C. An Infinite M200 Microplate Reader (TECAN, Switzerland) was used to detect absorbance at 490 nm.

### Colony-formation assay

Transfected and control cells were seeded in 6-well plates (1 × 10^3^ cells/well) and cultured for approximately 10 days. After three washings with cold PBS, the cells were fixed with cold methanol for 10 min, then stained with 0.1% crystal violet. Subsequently, the culture was washed with deionized water and photographed under a microscope.

### Wound-healing assay

Cells were scratched in 6-well plates with sterile 200-µL pipette tip to generate a straight line within each well. PBS was used to remove the detached cells. The cells were then cultured in medium containing 2% FBS for 20 h. The wounds in all experimental groups were monitored and photographed immediately (0 h) and 20 h after wound formation.

### Transwell-invasion assay

Matrigel was coated on polycarbonate inserts in a 24-well Boyden chamber (Corning, USA) to measure cell invasion. Cells were seeded in the upper chambers, and in the lower chambers 600 µL of culture medium containing 20% FBS was added to attract them chemically. After 20 h, cells on the underside of the membrane were washed gently with PBS, fixed for 10 min with methanol, and stained for 10 min with 0.5% crystal violet solution. Finally, the transwell chambers were washed with deionized water, the excess dye was removed, and images were captured under a microscope.

### Xenograft tumor model

5-week-old, female BALB/c nude mice were purchased from Vital River Laboratories (Beijing, China). The Institutional Animal Care and Use Committee of Peking University Cancer Hospital and Institute approved all animal experiments. We suspended 1 × 10^6^ KYSE30 cells stably overexpressing CPT1C and control cells in 100 µL PBS and injected the cells subcutaneously into the flanks of nude mice in separate groups (*n* = 6 mice/group). Following 5 weeks, the mice were sacrificed and tumor volumes calculated as follows: 0.5 × (length) × (width)^2^.

### Isolating mitochondria

Mitochondria isolation was performed using the Minute™ Mitochondria Isolation Kit (Invent Biotechnologies, USA). HeLa cells (3 × 10^7^) were collected to isolate intact mitochondria, according to the manufacturer’s protocol.

### Immunofluorescence

HeLa cells were seeded in glass-bottom dishes 1 day in advance of the immunofluorescence experiments. The cultured HeLa cells were fixed in cold methanol, permeabilized with 0.1% Triton X-100 for 5 min, blocked in 10% normal goat serum (ZSGB-BIO, China) for 40 min, incubated with the indicated primary antibodies at 4 °C overnight, washed, labeled with appropriate secondary antibodies for 1 h, and stained with DAPI for 5 min (1:10000, Beyotime, C1002). A Leica ST2 confocal microscope was used to acquire the images.

### Seahorse assays

Assays were performed using a Seahorse XF 96 analyzers (Agilent, USA) according to the manufacturer’s instructions. Concisely, 8 × 10^3^ cells/well were seeded in a 96-well XF cell culture microplate and incubated overnight in the recommended growth medium before the assay. The sensor cartridge was hydrated in a Seahorse XF Calibrant at 37 °C in a CO_2_-free incubator overnight. Subsequently, the medium was replaced with CO_2_-free low-buffered assay medium containing 2 mM L-glutamine (Lonza, 17-605E), 1 mM pyruvate (Sigma, S8636) and 25 mM glucose (Sigma, G7528). The oxygen-consumption rate (OCR) was measured at baseline and following sequential addition of 2 µM oligomycin (Abcam, ab141829), 1 µM FCCP (Sigma, C2920), 5 µM rotenone (Sigma, C2920), and 5 µM antimycin A (Abcam, ab141904). We used the total number of cells per well to normalize the OCR data.

### Intracellular ATP assay

Intracellular ATP levels were detected using an Enhanced ATP Assay Kit (Beyotime, S0027) following the manufacturer’s instructions. Transfected cells were lysed in lysis buffer on ice. Then, detection solution was added to a 96-well plate, and the lysates were incubated for 5 min at room temperature. The supernatants were then transferred into a 96-well plate and mixed quickly before luminescence signals were detected using an Infinite M200 Microplate reader (Tecan, Switzerland). The resulting data were normalized to the protein concentrations in each sample, and relative ATP levels were calculated based on chemiluminescence absorbance.

### Intracellular ROS assay

Intracellular ROS levels were measured using a Reactive Oxygen Species Assay Kit (Beyotime, S0033S) following the manufacturer’s instructions. We added DCFH-DA (Beyotime, S0033M-1) to the medium at a final concentration of 10 µM. After adding DCFH-DA, the cells were incubated at 37 °C for 20 min, with mixing by inversion every 3–5 min. Then, the cells were washed and collected for analysis with the Accuri C6 flow cytometer (BD Biosciences). The mean fluorescence intensities were used to calculate the ROS contents.

### Immunohistochemical (IHC) staining

Tissues were fixed, deparaffinized, and hydrated, and their antigens were retrieved using EDTA antigenic retrieval buffer by boiling the tissues in a pressure cooker for 10 min. Then, the slides were blocked using 10% goat serum (ZSGB-BIO, China) for 40 min at room temperature, labeled overnight at 4 °C with an anti-CPT1C antibody (1:400, Proteintech, 66072-1-Ig) and an anti-Ki-67 antibody (1:200, Abcam, ab15580), tagged with a universal secondary antibody (ZSGB-BIO, PV-6000) for 40 min at room temperature, and stained with 1× DAB solution (ZSGB-BIO, ZLI-9019).

A tissue microarray was constructed using tumor tissues from 102 patients with primary ESCC who underwent surgery between February, 2011 and October, 2013, which was purchased from Shanghai Outdo Biotech Co., Ltd. (Shanghai, China). To conduct IHC analysis of the tissue microarray, tissue specimens were subjected to immunostaining using an anti-CPT1C antibody (1:400, Proteintech, 66072-1-Ig). The Institutional Research Ethics Committee provided ethical approval and documented informed consent for all patients. Supplementary Table S2 displays the detailed clinicopathological characteristics of all specimens. The specimens were assigned the following staining-intensity scores: 0, 0.5, 1, 2, and 3. The positive-staining rates varied between 0% and 100%. The total staining score was calculated as the product of the staining-intensity score and the staining-positivity rate, resulting in values ranging from 0 to 300%. Based on the median IHC score, the ideal cut-off point was 0.1485. An IHC score for CPT1C staining of > 1.485 was identified as high expression and a score of ≤ 1.485 was identified as low expression.

### Statistical analysis

Statistical analyses were performed using SPSS version 27.0 (IBM) and GraphPad Prism 8. A two-tailed Student’s *t*-test was used to assess the statistical significance of comparisons between the control and treatment groups. Two-tailed Pearson’s correlation coefficients were used to assess correlations between CPT1C expression and clinicopathological parameters. Overall survival (OS) curves were constructed based on Kaplan-Meier survival analysis and the log-rank test. Survival data were also evaluated using multivariate Cox regression analysis. The results are presented as mean ± standard error of the mean (SEM) of values obtained from at least three independent experiments. *P* < 0.05 was considered to reflect a statistically significant difference.

## Results

### CPT1C expression was cell cycle-dependent manner and peaked at the G1/S boundary

**Fig. 1:**
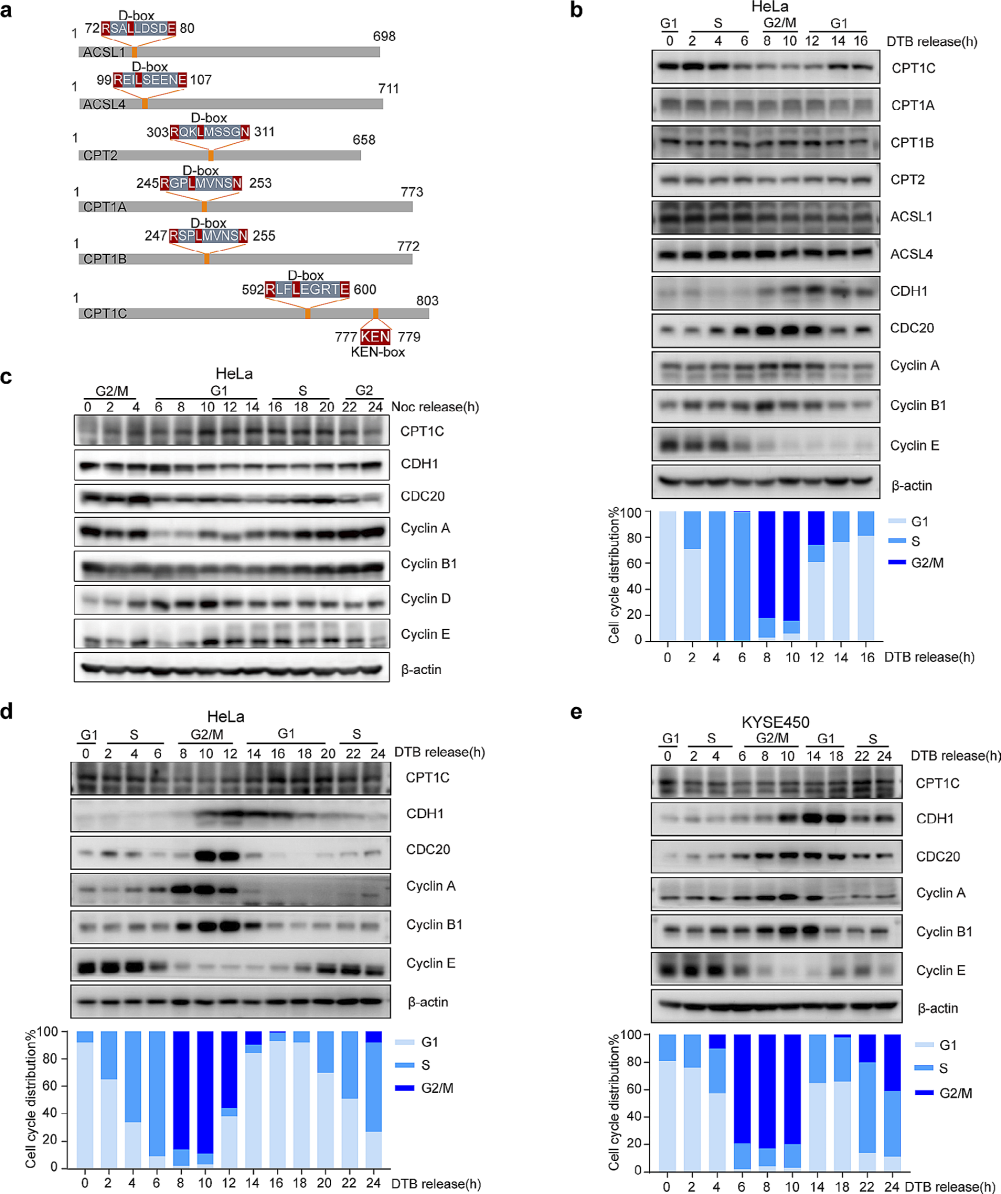
CPT1C was expressed in a cell cycle-dependent manner and peaked at the G1/S boundary. (**a**) Schematic illustration of multiple FAO-related enzymes. (**b**) HeLa cells were arrested at the G1/S boundary by DTB treatment, released into fresh medium, and harvested at the indicated time points. In the upper panel, cells were collected for immunoblotting. In the bottom panel, cells were stained with PI and analyzed by flow cytometry. (**c**) HeLa cells were arrested at the G2/M phase by nocodazole treatment, released into fresh medium, and harvested at the indicated time points for immunoblotting. (**d**) HeLa cells were arrested at the G1/S boundary by DTB, released into fresh medium, and harvested at the indicated time points for immunoblotting (upper panel) and flow cytometry (bottom panel). (**e**) KYSE450 cells were arrested at the G1/S boundary by DTB treatment, released into fresh medium, and harvested at the indicated times for immunoblotting (upper panel) and flow cytometry (lower panel). Three independent experiments were performed. DTB, double thymidine block; Noc, nocodazole

Given that previous research has established the role of the APC/C in regulating multiple metabolic networks, we aimed to explore whether the APC/C might be involved in fatty acid oxidation. Initially, we analyzed the amino acid sequences of several FAO-related enzymes and found that CPT1A, CPT1B, CPT2, acyl-CoA synthetase long-chain family member 1 (ACSL1), and ACSL4 contained a D-box, whereas CPT1C possessed both D-box and KEN-box sequences (Fig. 1a). To our knowledge, most APC/C-regulated proteins exhibit cell cycle-dependent expression [33]. Thus, we investigated whether their expression levels were associated with cell cycle progression. We synchronized HeLa cells at the G1/S boundary using a DTB and then released the block by switching the cells to fresh medium. Cells harvested at the indicated time points were subjected to immunoblotting and flow cytometric analysis based on propidium iodide (PI) staining. The cyclins expression levels and PI-staining profiles were used to distinguish between distinct cell cycle phases. Our results indicated that CPT1C protein levels fluctuated markedly in a cell cycle-dependent manner, peaking during the G1/S transition and gradually decreasing until G2/M phase (Fig. 1b; Supplementary Fig. S1a). Nevertheless, other FAO-related enzymes, including CPT1A, CPT1B, CPT2, ACSL1, and ACSL4 showed no obvious variations (Fig. 1b). These preliminary results suggest that CPT1C expression was regulated by cell cycle regulators. To confirm that CPT1C was cell cycle-dependent, we employed different cell-synchronization methods and extended the release time to 24 h, covering the entire cell cycle. We synchronized HeLa cells in G2/M phase via nocodazole treatment and observed that the CPT1C protein abundance was highest in late G1 phase and early S phase (Fig. 1c). Similar oscillations in CPT1C expression were detected in double thymidine-treated HeLa cells, in which CPT1C was highly expressed in G1/S phase (Fig. 1d and Supplementary Fig. S1b). This phenomenon was further confirmed in KYSE-450 cells (Fig. 1e; Supplementary Fig. S1c) and KYSE-510 cells (Supplementary Fig. S1d, e). Taken together, our results show that CPT1C expression was cell cycle-dependent and that it peaked at the G1/S boundary.

### CPT1C protein levels were regulated by the APC/C-CDH1 and APC/C-CDC20 complexes

**Fig. 2:**
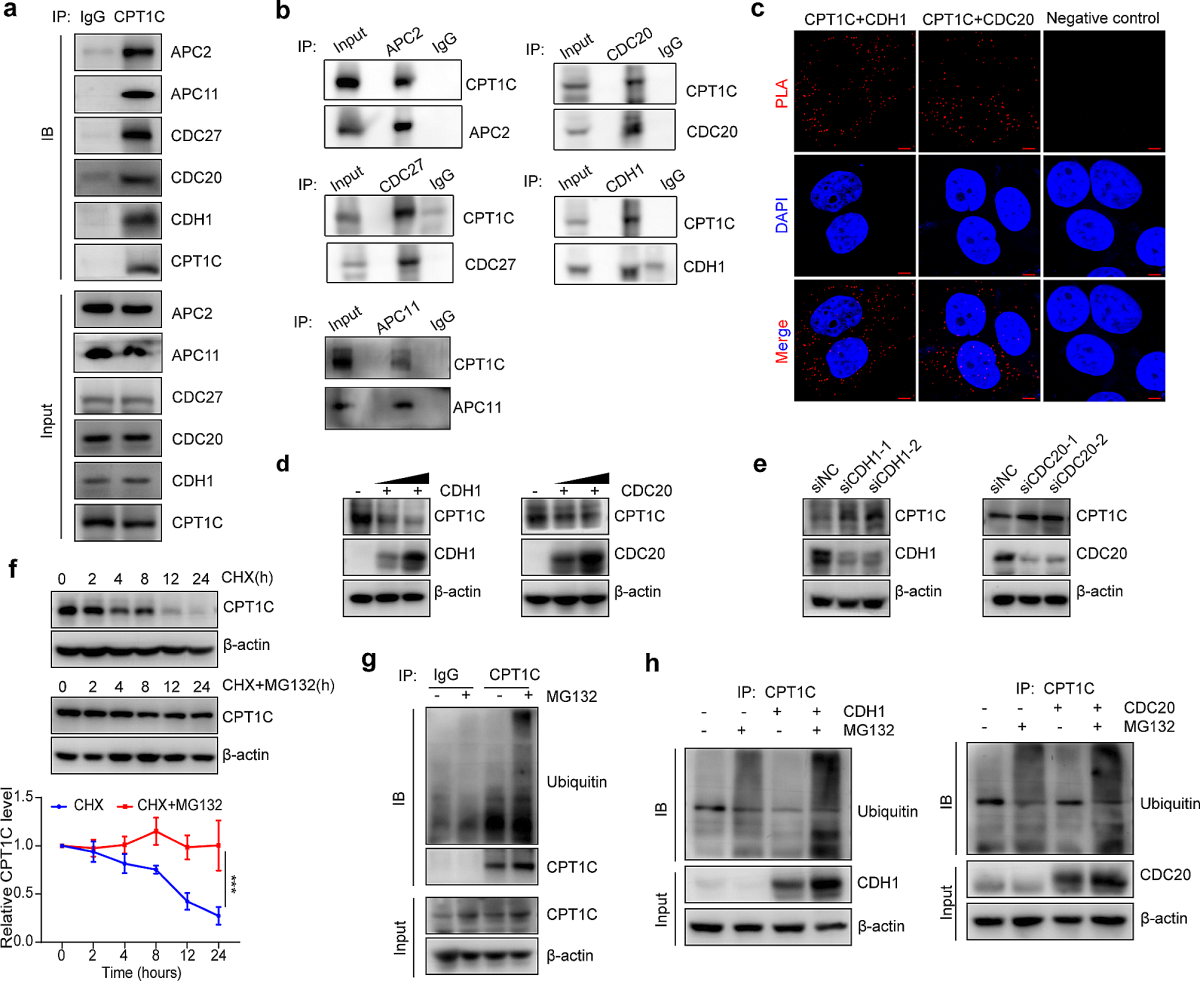
CPT1C protein levels were regulated by the APC/C-CDH1 and APC/C-CDC20 complexes. (**a**) CPT1C was immunoprecipitated using anti-APC2, anti-APC11, anti-CDC27, anti-CDC20, and anti-CDH1 antibodies. (**b**) HeLa cell lysates were immunoprecipitated with anti-APC2, anti-CDC27, anti-CDC20, and anti-CDH1 antibodies to detect CPT1C binding. (**c**) PLAs were performed between endogenous CPT1C, CDC20, and CDH1 in HeLa cells, as well as anti-IgG as a negative control. Scale bar: 5 μm. (**d**) HeLa cells were transfected with a CDH1 (left) or CDC20 (right) plasmid, and CPT1C protein levels were detected by immunoblotting. (**e**) HeLa cells were transfected with siCDH1(left) or siCDC20 (right). and CPT1C protein levels were detected by immunoblotting. (**f**) HeLa cells were treated with 100 µg/mL CHX or CHX with 20 µM MG132 for the indicted time, and CPT1C proteins levels were detected by western blot analysis. Quantification of CPT1C expression is shown in the lower panel. (**g**) HeLa cells were incubated with (+) or without (-) 20 µM MG132 for 6 h before harvesting. Lysates were subjected to IP assays with an anti-CPT1C antibody or anti-IgG as a negative control, followed by immunoblotting to detect the ubiquitination level of CPT1C. (**h**) HeLa cells were transfected with CDH1 or CDC20 plasmid for 48 h, incubated with 20 µM MG132 for 6 h, and subjected to IP and subsequent immunoblotting analysis. Three independent experiments were performed. The error bars represent the mean ± SEM. ***P < 0.001; two-tailed unpaired Student’s *t*-test

To verify that CPT1C is a novel APC/C substrate, we performed co-IP experiments and discovered that CPT1C interacts with three essential components of APC/C [34]: APC2, APC11 and CDC27 (Fig. 2a, b). Moreover, we conducted PLA and co-IP assays, which demonstrated that CP1TC directly interacted with CDH1 and CDC20, two APC/C coactivators (Fig. 2a-c). Since APC/C, along with its coactivators, serves as an E3 ubiquitin ligase, we hypothesized that CPT1C would be affected by these coactivators at the post-translational level. Thus, we overexpressed CDH1 and CDC20 separately in HeLa cells and observed that the CPT1C protein levels decreased significantly (Fig. 2d). Conversely, CPT1C protein levels increased when CDH1 or CDC20 were knocked down (Fig. 2e). Next, we sought to confirm whether CPT1C is degraded via the ubiquitin-proteasomal pathway. We treated HeLa cells with a protein-synthesis inhibitor, cycloheximide (CHX), or CHX combined with the proteasome inhibitor, MG132. As expected, MG132 stabilized the CPT1C protein-expression level (Fig. 2f) and led to a significant increase in CPT1C ubiquitylation, as observed in ubiquitin pull-down assays (Fig. 2g). We also found that ubiquitinated CPT1C expression increased in CDH1-overexpressing cells and CDC20-overexpressing cells and that this elevation was particularly prominent after MG132 treatment (Fig. 2h). Overall, these results indicate that CPT1C protein levels were regulated by the APC/C-CDH1 and APC/C-CDC20 complexes.

### The D-box and KEN box were essential for ubiquitin-dependent CPT1C degradation

**Fig. 3:**
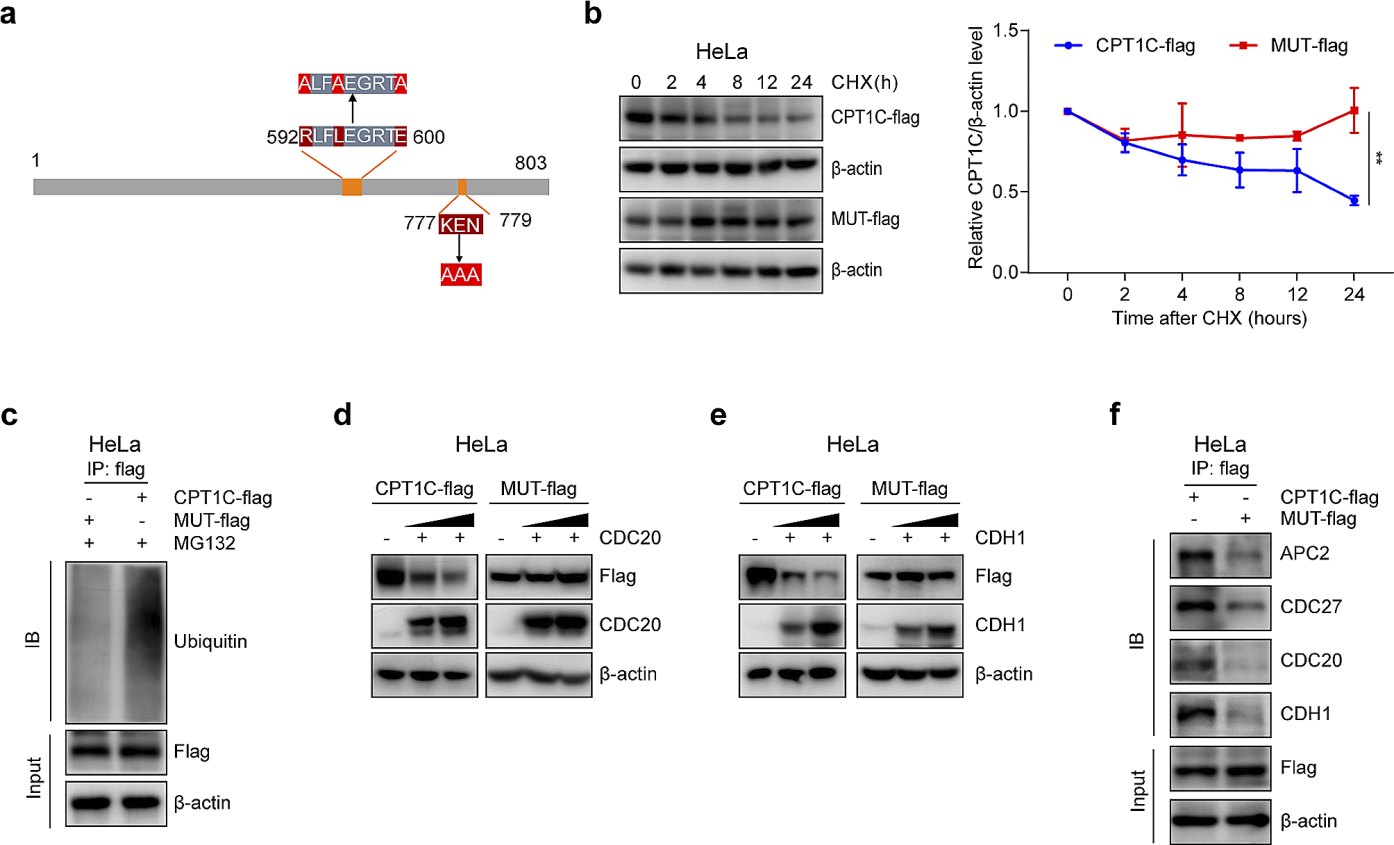
The D-box and KEN-box were essential for ubiquitin-dependent CPT1C degradation. (**a**) Schematic representation of mutant CPT1C (MUT-flag). The numerals indicate the amino acid positions at which the consensus sequence starts, relative to the initiation codon. (**b**) CPT1C-flag and MUT-flag plasmids were transfected into HeLa cells. The HeLa cells were treated with 100 µg/mL CHX and harvested at the indicated time points for immunoblotting. Quantification of exogenous CPT1C expression is shown in the right panel. (**c**) HeLa cells were transfected with a CPT1C-flag or MUT-flag plasmid for 48 h and then incubated with 20 µM MG132 for 6 h before harvest. Exogenous wild-type CPT1C and mutated CPT1C were immunoprecipitated from whole-cell extracts. (**d**) HeLa cells were co-transfected with a CPT1C-flag or MUT-flag plasmid and different concentrations of a CDC20 plasmid. Cells were lysed after 48 h of transfection and exogenous CPT1C and CDC20 protein levels were evaluated by immunoblotting. (**e**) HeLa cells were co-transfected with CPT1C-flag or MUT-flag and different concentrations of the CDH1 plasmid. Cells were lysed after 48 h of transfection, and exogenous CPT1C and CDH1 protein levels were evaluated by immunoblotting. (**f**) IP assays were performed using HeLa cell extracts with ectopic expression of CPT1C-flag or MUT-flag to compare their binding to APC2, CDC27, CDH1, and CDC20. Three independent experiments were performed. The error bars represent the mean ± SEM. **P < 0.05; two-tailed unpaired Student’s *t*-test

To further determine whether the D-box and KEN-box were responsible for the oscillations in CPT1C expression, site mutations were introduced into the KEN box and D-box of wild-type CPT1C (Fig. 3a). Subsequently, exogenous wild-type and mutant CPT1C were detected after different incubation times following a chase with CHX. Notably, the half-life of the mutant CPT1C protein appeared to be longer than that of the wild-type CPT1C protein (Fig. 3b), whereas the ubiquitylation level of the mutant CPT1C was attenuated, as expected (Fig. 3c). In contrast to wild-type CPT1C, the protein levels of mutant CPT1C were not affected by CDC20 and CDH1 overexpression (Fig. 3d, e). Furthermore, we conducted co-IP experiments to assess the binding affinities of wild-type and mutant CPT1C to APC/C. We compared with wild-type CPT1C, the mutant CPT1C displayed significantly weaker binding to APC2, CDC27, CDH1, and CDC20 (Fig. 3f). Collectively, our data suggest that both conserved motifs, i.e., the D-box and KEN box, were required for APC/C-mediated ubiquitin-dependent CPT1C degradation.

### CPT1C accelerated the G1/S transition and promoted the malignant proliferation of tumor cells

**Fig. 4:**
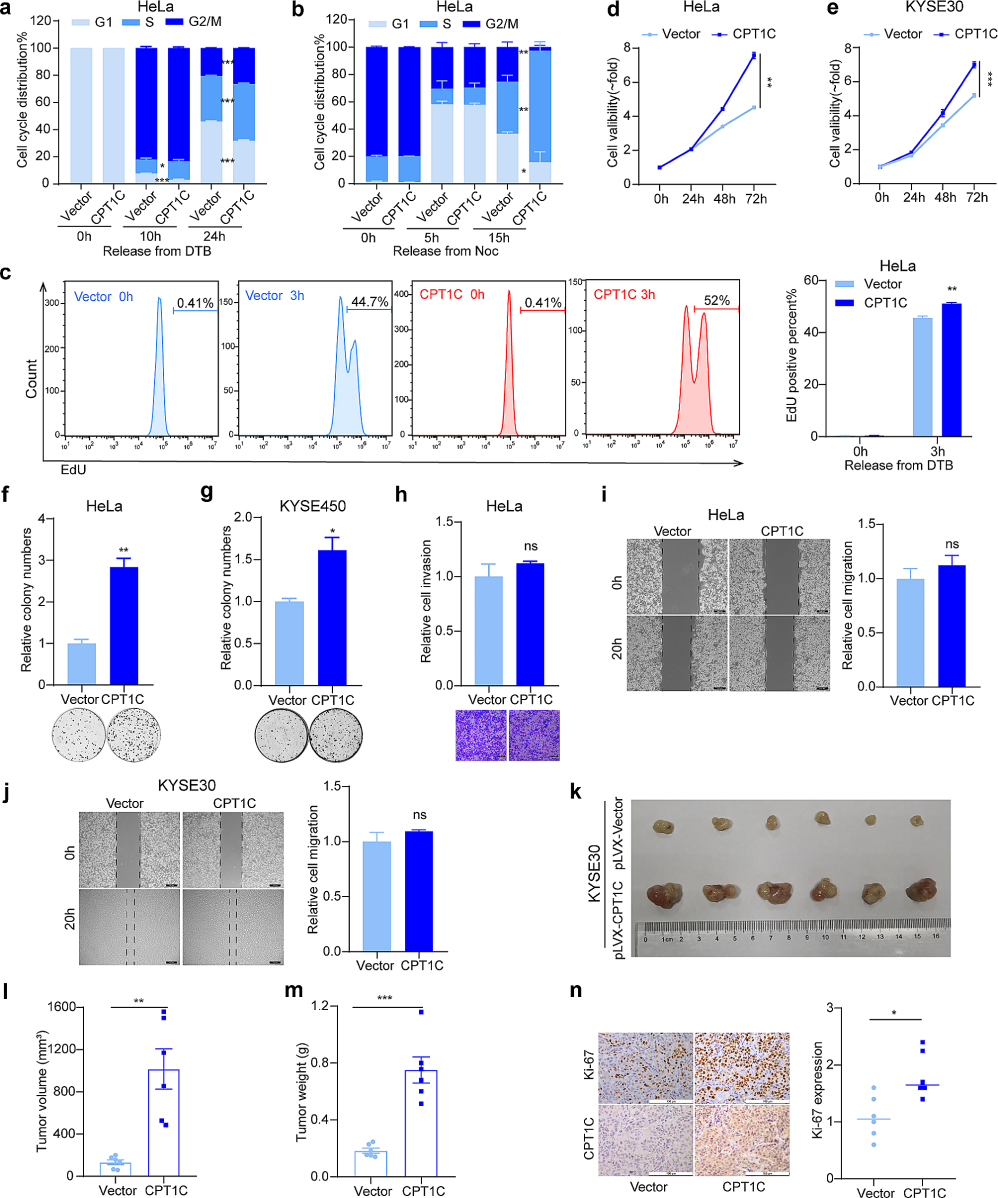
CPT1C accelerated the G1/S transition and promoted the malignant proliferation of tumor cells. (**a**, **b**) Cell cycle analysis of HeLa cells by flow cytometry at the indicated time points after release from DTB treatment (**a**) and Noc treatment (**b**). (**c**) Flow cytometry-based EdU-incorporation assays were performed with HeLa cells 3 h after release a DTB (left) and quantitation of the percentages of cells that entered S phase (right). (**d**, **e**) MTS assay showing the viability of HeLa (**d**) and KYSE30 cells (**e**) transfected with empty vectors or CPT1C plasmids at the indicated times. (**f**) and (**g**) Colony-formation assays performed with HeLa cells (**f**) and KYSE450 cells (**g**) transfected with empty vectors or CPT1C plasmids. Representative images (bottom panel) and quantitative results (top panel). (**h**) Transwell assay data showing the viability of HeLa cells transfected with empty vectors or CPT1C plasmids. Representative images (left) and quantitative results (right). Scale bar: 100 μm. (**i**, **j**) Scratch-wound assays showing cell mobility of HeLa cells (**i**) and KYSE30 cells (**J**) transfected with empty vectors or CPT1C plasmids. Representative images (left) and quantitative results (right). Scale bar: 100 μm. (**k-m**) Image (**k**), volume (**l**), and weight (**m**) of xenograft tumors derived from control KYSE30 cells and KYSE30 cells stably overexpressing CPT1C (*n* = 6). (**n)** Ki-67 staining of CPT1C-overexpressing tumor tissues and control tissues. Representative images (left) and quantitative results (right). Scale bar: 100 μm. Three independent experiments were performed. The error bars represent the mean ± SEM. *P < 0.05; **P < 0.01; ***P < 0.001; ns, not statistically significant; two-tailed unpaired Student’s *t*-test; Noc, nocodazole; DTB, double thymidine block

Precise G1/S transition is crucial for controlling cell proliferation, and its dysregulation leads to genomic instability and oncogenesis [35]. We investigated the effect of CPT1C on the G1/S transition since the above results indicated CPT1C accumulated in late G1 phase and early S phase. After HeLa cells were released from synchronization, we analyzed the cell cycle distribution by flow cytometry at the indicated time points. When the cells were released from the G1/S boundary, the percentage of cells in S phase in CPT1C-overexpressing cells was markedly higher than that in control cells (Fig. 4a; Supplementary Fig. S2a, b; Supplementary Table S3). Similarly, we detected a higher percentage of S-phase cells in CPT1C-overexpressing cells released from G2/M synchronization (Fig. 4b; Supplementary Fig. S2c; Supplementary Table S4). These results indicate that CPT1C-overexpressing cells entered S phase more quickly than control cells. EdU is a thymidine analog that is only incorporated into cells during S phase; hence, it is used to evaluate the proportion and proliferation of cells in S phase [36]. HeLa cells were released from the G1/S boundary and incubated with EdU. The percentage of EdU-positive cells was higher in CPT1C-overexpressing cells than in control cells (Fig. 4c). These results further suggest that CPT1C promoted the G1/S transition.

Given that CPT1C promoted the G1/S transition, we explored the biological function of CPT1C in malignant tumor progression by measuring the proliferative ability of tumor cells in MTS assays. CPT1C-overexpressing tumor cells exhibited a higher rate of proliferation than control cells (Fig. 4d, e; Supplementary Fig. S2d). Next, we conducted colony formation assays and observed more colonies with CPT1C-overexpressing cells than in the control group (Fig. 4f, g; Supplementary Fig. S2e). In addition, we evaluated the impact of CPT1C on cell invasion and migration in vitro by performing matrigel-based invasion assays (Fig. 4h) and wound-healing assays (Fig. 4i, j). We found that CPT1C did not significantly affect cell mobility or invasiveness. To further demonstrate the role of CPT1C in tumor promotion, we subcutaneously injected KYSE30 cells stably overexpressing CPT1C or control cells into nude mice (Supplementary Fig. S2f). The xenograft tumors in the CPT1C-overexpression group were heavier with larger volumes than those in the control group (Fig. 4k-m). Moreover, increased Ki-67 expression (as detected by IHC staining) was observed in the CPT1C-overexpressing group (Fig. 4n). These results indicate that CPT1C promoted the G1/S transition and malignant proliferation of tumor cells in vitro and in vivo.

### CPT1C promoted mitochondrial respiratory productivity and maintained redox homeostasis

**Fig. 5:**
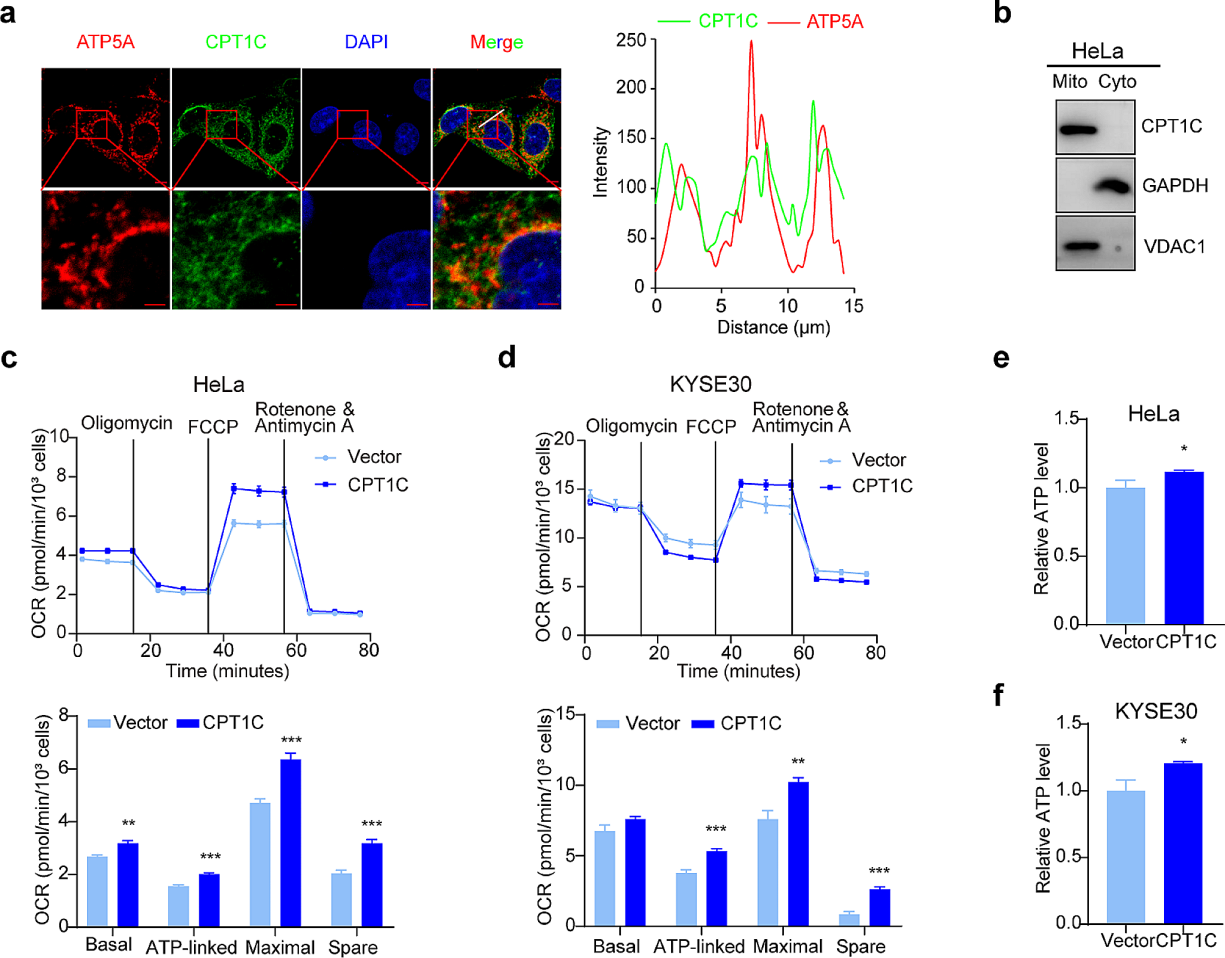
CPT1C promoted mitochondrial respiratory productivity. (**a**) Immunofluorescence analysis showing the colocalization of CPT1C and ATP5A in HeLa cells. The white lines indicate fluorescence-intensity line plots. Scale bar: 5 μm (upper), 2 μm (lower). (**b**) Lysates from the cytoplasmic and mitochondrial fractions were analyzed by immunoblotting. (**c**, **d**) OCRs were measured in control or CPT1C-overexpressing HeLa cells (**c**) and KYSE30 cells (**d**). The OCR trace is presented in the upper panel. The basal, ATP-linked, maximal, and sparse OCR analysis results are shown in the bottom panel. (**e**, **f**) ATP assays were performed to evaluate the intracellular ATP levels in control or CPT1C-overexpressing HeLa cells (**e**) and KYSE30 cells (**f**). Three independent experiments were performed. The error bars represent the mean ± SEM. *P < 0.05; **P < 0.01; ***P < 0.001; two-tailed unpaired Student’s *t*-test

To investigate whether CPT1C promoted cell proliferation in a manner related to energy metabolism, we performed immunofluorescence staining in HeLa cells to investigate the localization of CPT1C. Immunofluorescence analyses revealed co-localization between CPT1C and ATP5A, a mitochondrial marker [37], within the mitochondria (Fig. 5a). Furthermore, we isolated mitochondria for immunoblotting. CPT1C was detected in the mitochondrial components, voltage-dependent anion channel 1 (VDAC1) served as a specific marker for the mitochondrial fraction [38] and GAPDH was used as a cytoplasmic marker (Fig. 5b). Next, we assessed the impact of CPT1C on oxidative phosphorylation in tumor cells using a Seahorse XF96 extracellular flux analyzer. When compared with the control groups, CPT1C showed a higher OCR in HeLa cells (Fig. 5c) and KYSE30 cells (Fig. 5d), as assessed by measuring basal respiration, ATP-linked respiration, maximum respiration, and the spare respiratory capacity. Moreover, we measured cellular ATP levels and observed that they were elevated in CPT1C-overexpressing HeLa cells (Fig. 5e) and KYSE30 cells (Fig. 5f).

**Fig. 6:**
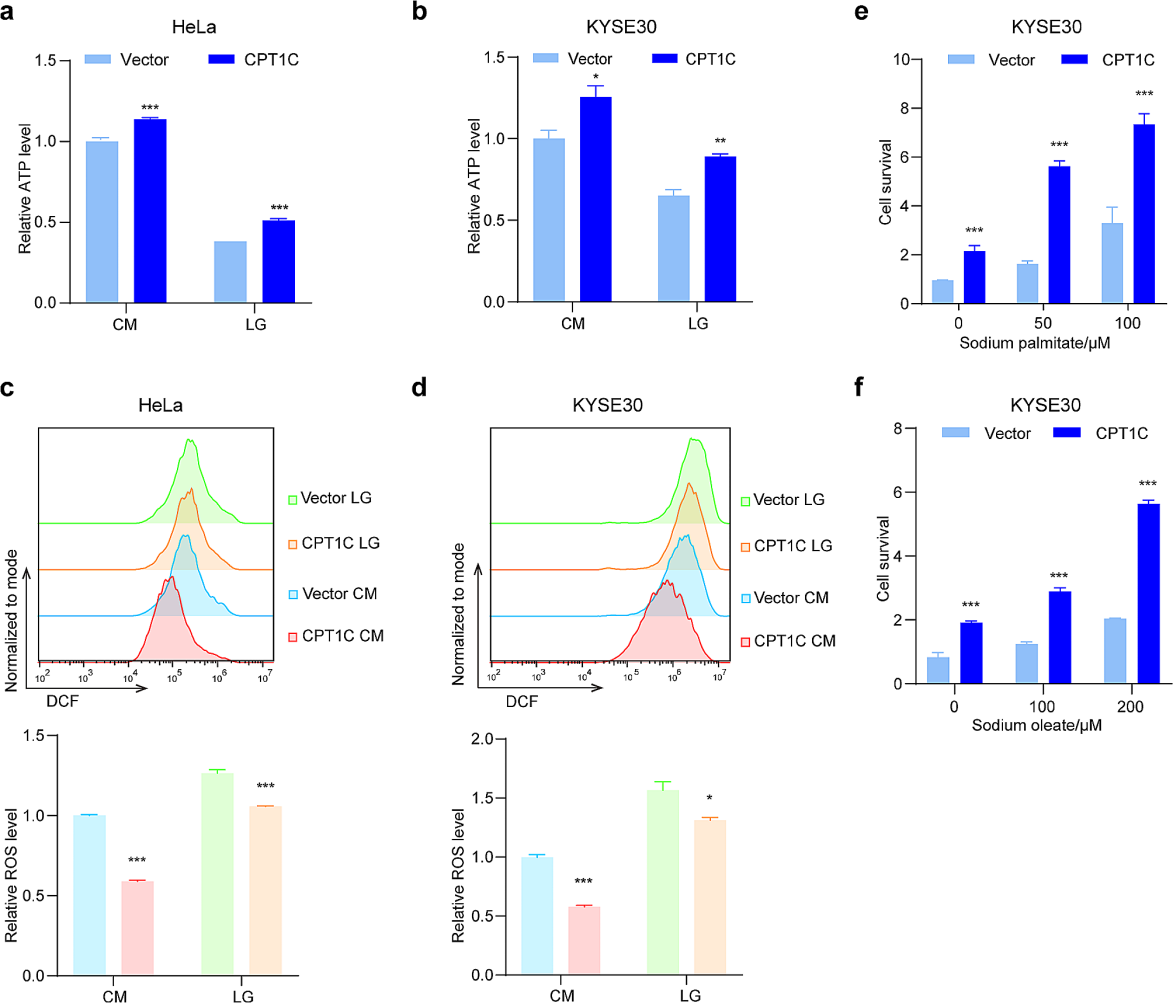
CPT1C promoted tumor cell survival with low glucose by upregulating energy and maintaining redox homeostasis. (**a**, **b**) ATP assays were performed to evaluate intracellular ATP levels in control or CPT1C-overexpressing HeLa cells (**a**) and KYSE30 cells (**b**) in low-glucose (LG) or control medium (CM). (**c**, **d**) Flow cytometry was performed to evaluate the intracellular ROS levels in control or CPT1C-overexpressing HeLa cells (**c**) and KYSE30 cells (**d**) under LG or control conditions. Quantification of the DCFH-DA fluorescence intensity, which indicates the relative intracellular ROS levels, is shown in the right panel. (**e**, **f**) Under the LG condition, MTS assays were performed with control or CPT1C-overexpressing KYSE30 cells after incubation with the indicated concentrations of sodium palmitate (**e**) and sodium oleate (**f**) for 48 h. Three independent experiments were performed. The error bars represent the mean ± SEM. *P < 0.05; **P < 0.01; ***P < 0.001; two-tailed unpaired Student’s *t*-test

An important aspect of tumor metabolic reprogramming is the utilization of various nutrients to favor cell proliferation and survival. Tumor cells utilize various nutrient sources to meet anabolic needs and maintain energy and redox homeostasis, especially under nutrient-restricted conditions [39–41]. Hence, we explored the influence of CPT1C on tumor cells cultured in medium containing 5 mM glucose to mimic the nutrient-restricted microenvironment, whereas the control medium contained 11 mM glucose. The results showed that CPT1C overexpression contributed to increased ATP levels in HeLa (Fig. 6a) and KYSE30 cells (Fig. 6b), while significantly decreasing reactive oxygen species (ROS) levels in HeLa (Fig. 6c) and KYSE30 cells (Fig. 6d). To determine whether CPT1C could facilitate tumor cells to utilize free fatty acids for survival in nutrient-restricted conditions, KYSE30 cells were maintained in medium containing 5 mM glucose added with varying concentrations of free fatty acids, and cell viabilities were measured using the MTS assay. Forced CPT1C expression enhanced the utilization of free fatty acids and facilitated tumor cell survival under low-glucose conditions (Fig. 6e, f). These results strongly suggest that CPT1C facilitated ATP production to alleviate low glucose-induced metabolic stress, maintain cellular energy and redox homeostasis, and ultimately promoted tumor cell survival.

### CPT1C was significantly upregulated in ESCC tumors and correlated with a poor prognosis

**Fig. 7:**
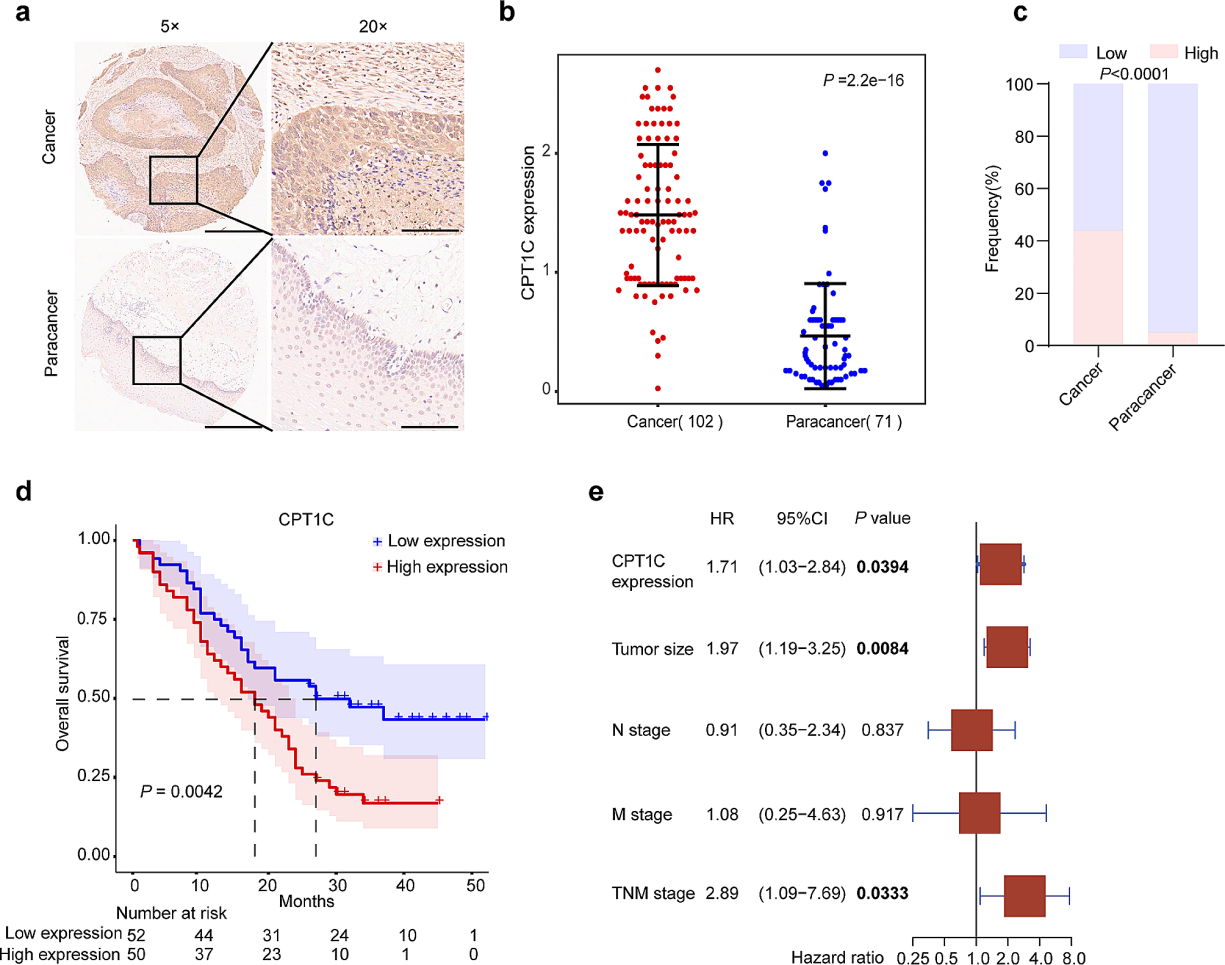
CPT1C was significantly upregulated in ESCC tumor tissues, which correlated with a poor prognosis. (**a**) Representative IHC-staining image for CPT1C in ESCC and adjacent normal tissues. Scale bar: 500 μm (left), 125 μm (right). (**b**) Statistical analysis of CPT1C expression levels in ESCC and adjacent normal tissues (Wilcoxon test, ***P < 0.001). (**c**) Proportions of tumor and adjacent normal tissues with low or high CPT1C expression (Fisher’s exact test, ***P < 0.001). (**d**) Kaplan-Meier survival analysis revealing an association between the OS time and CPT1C protein levels in patients with ESCC (log-rank test, **P < 0.01). (**e**) Multivariate Cox regression analysis of the associations between survival, CPT1C protein levels, and clinicopathological factors. Hazard responses (HRs) and 95% confidence intervals (CIs) were plotted for each variable

To investigate the clinical significance of CPT1C expression, we performed IHC staining to detect CPT1C levels in a tissue microarray comprising 102 ESCC tissues and 71 paired adjacent normal tissues. CPT1C expression was scored based on the positive-staining intensity and ratio of CPT1C-positive cells. Tumor tissues exhibited relatively strong positive CPT1C expression, whereas most paired adjacent normal tissues showed weakly positive or negative expression (Fig. 7a). Statistical analysis demonstrated that a notable increase in CPT1C expression in ESCC tissues compared to the adjacent normal tissues (*P* = 2.2e-16, Fig. 7b). The samples were classified as CPT1C-high or CPT1C-low expression, and a significant difference was observed in the distributions of samples between tumor tissues and adjacent normal tissues (Fig. 7c; Supplementary Table S5). Importantly, the median OS of the group with high CPT1C expression was 18 months, whereas that of the group with low CPT1C expression was 27 months. Kaplan-Meier survival analysis showed that the overall survival time for patients with high CPT1C expression was significantly shorter than that for patients with low CPT1C expression (*P* = 0.0042; Fig. 7d). We further explored the association between CPT1C expression and the clinicopathological features of patients with ESCC. The expression level of CPT1C did not correlate significantly with the following clinical features: sex, age, tumor size, lymph node, metastasis, grade, or tumor-node-metastasis (TNM) stage (*P* > 0.05; Supplementary Table S2). Multivariate Cox regression-survival analysis (adjusted for the tumor size, N stage, M stage, TNM stage, and CPT1C level) revealed a strong correlation between high CPT1C expression and a shorter OS (*P* = 0.0394, HR = 1.71, 95% CI, 1.03–2.84, Fig. 7e; Supplementary Table S6) and that CPT1C expression was an independent prognostic indictor for patients with ESCC. Overall, these findings powerfully imply that CPT1C was upregulated in ESCC and that it may serve as a prognostic marker.

## Discussion

Our findings demonstrate that CPT1C is a novel APC/C substrate and is expressed in a cell cycle-dependent manner, revealing a new form of cross-talk between fatty acid utilization and the cell cycle. Here, we not only demonstrated that APC/C regulates CPT1C expression, but we also validated the ability of CPT1C to accelerate the G1/S transition, resulting in increased cell proliferation in vitro and in vivo. These finds reveal a new bidirectional regulation between the cell cycle and fatty acid utilization. Furthermore, CPT1C overexpression promotes mitochondrial energy production, resistance to metabolic stress, and the maintenance of redox homeostasis. Importantly, high CPT1C-expression levels are associated with poor survival in patients with ESCC, indicating that CPT1C-dependent metabolic alterations promote ESCC progression and that CPT1C is a promising prognostic indicator.

The demands for biosynthesis and energy vary in different phases of the cell cycle; therefore, metabolic activities exhibit cell cycle-dependent fluctuations [42–44]. Contrarily, the cell cycle machinery is responsible for regulating metabolic pathways to ensure that cells generate sufficient biomass and the energy required for proliferation [45, 46]. APC/C plays crucial roles in linking the cell cycle to metabolic activities by mediating degradation of various metabolic enzymes via ubiquitination modification. In late G1 phase, inactivation of the APC/C-CDH1 complex leads to PFKFB3 accumulation, which in turn promotes glycolysis and proliferation [47, 48]. Furthermore, APC/C directs the degradation of GLS1 during mitotic exit and G1 phase, ensuring that GLS1 remains highly activated and enhances glutaminolysis during S phase to support DNA replication [7, 11]. Additionally, our previous findings have also displayed the regulation of APC/C on IDH3β involving in TCA cycle [13]. Further studies are necessary to determine whether APC/Cs are involved in regulating lipid metabolism.

Tumor cells reprogram their metabolism for greater flexibility, which enables rapid growth and an enhanced ability to pass through cell cycle checkpoints [49]. Apart from glucose, fatty acids are important energy sources. In recent years, fatty acid metabolism in tumor cells has attracted increasing attention. Prostate cancer cells use β-oxidation of fatty acids as their main energy source [50], and ACSL1 promotes prostate cancer progression by increasing lipogenesis and FAO [51]. CPT1A is highly expressed in ovarian cancer and upregulated FAO mediated by CPT1A to promote ovarian cancer progression [52]. CPT1B plays critical roles in maintaining breast cancer cell stemness and enhancing chemoresistance [24]. Given the importance of FAO-related enzymes, we analyzed the amino acid sequences of multiple FAO-related enzymes and found that only CPT1C contained both a D-box and a KEN-box. We verified that CPT1C is indeed a new APC/C substrate regulated by the APC/C-CDC20 and APC/C-CDH1 complexes. CPT1C protein levels accumulated during the late G1 and early S phases. Hence, our results indicated a novel connection between APC/C and CPT1C, a rate-limiting enzyme in FAO.

The other two members of the key FAO-related CPT1 enzyme family are CPT1A and CPT1B. Although the nucleotide sequence of the coding region of CPT1C shares 86% and 85% identity with those of CPT1A and CPT1B, respectively, they show differences in terms of tissue distribution, subcellular localization, and biological function [26]. CPT1A is primarily distributed in the liver, whereas CPT1B is primarily distributed in the heart and skeletal muscles. CPT1C is expressed predominantly in the mammalian brain. However, the expression of these CPT1 family members is usually dysregulated in tumor tissues [53]. The consensus view is that both CPT1A and CPT1B are located in the mitochondria [54], but the subcellular localization of CPT1C is controversial. Several findings have shown that CPT1C is localized in the endoplasmic reticulum of stem cells and neurons but not in the mitochondria [55, 56]. CPT1C has also been detected in mitochondria-associated membranes and microsomes in MCF7 cells [57]. Our data indicated that CPT1C was present in mitochondria in HeLa cells. Hence, the discrepancy in CPT1C localization might be attributed to difference among cell lines. However, we did not conduct experiments to exclude CPT1C localization to other subcellular structures. Moreover, we observed that only CPT1C was regulated by the core cell machinery, APC/C, whereas CPT1A and CPT1B were not. CPT1A and CPT1B have similar functions, and the results of many studies have shown that FAO is upregulated under normal glucose conditions [24, 52, 58]. We also found that CPT1C enhanced fatty acid utilization and promoted ESCC cell survival under low-glucose conditions. These results inspired us to explore why only CPT1C exhibited cell cycle-dependent expression. During the restriction period in late G1 phase, which is highly nutrient-sensitive, the G1/S transition requires an energy boost [2, 7]. Our results demonstrated that CPT1C protein levels peaked at the G1/S boundary and that CTP1C overexpression accelerated the G1/S transition. Accordingly, we speculate that CPT1C functions as a compensatory mechanism with other two family members, particularly playing a crucial role during the G1/S transition phase. Moreover, CPT1C facilitated fatty acid utilization and survival in harsh environments. Therefore, cells greatly require certain proteins, such as CPT1C, which are instrumental in enabling cells to withstand metabolic stress during this nutrient-sensitive period.

An altered redox status is a frequently observed hallmark of tumor cells [59]. Generally, a moderate level of ROS favors tumor cell proliferation and survival, whereas excessive ROS usually causes oxidative stress and cell death [59]. Glucose deprivation and glycolysis inhibition induce excessive ROS production and decrease ATP production in glioma, breast, and colon cancer cells [60, 61]. To adapt to harsh environments, solid tumor cells optimize nutrient utilization to fulfill their energy requirements and maintain redox homeostasis [39–41]. In this study, we cultured ESCC KYSE30 cells in a low-glucose medium with or without added fatty acids. CPT1C overexpression increased ATP production and decreased ROS levels in a concentration-dependent manner. Here, we observed for the first time that CPT1C promoted cell survival by alleviating low glucose-induced metabolic stress and maintaining cellular energy and redox homeostasis in ESCC cells. However, we have not delved into the upstream factors that drive CPT1C to function under metabolic stress. The precise molecular mechanisms whereby CPT1C overcomes metabolic stress require further elucidation. Zaugg K [62] reported that CPT1C is a target gene of p53, and under metabolic stress conditions, p53-dependent AMPK can induce the expression of CPT1C to help cells resist metabolic stress. In the HeLa cells, KYSE30 cells, KYSE450 cells, and KYSE510 cells used in this study, p53 is either mutated or functionally deficient, implying the potential for alternative upstream mechanisms that may modulate CPT1C expression in a p53-independent manner. This observation underscores the complex regulatory networks within cancer cells, which employ diverse strategies to adapt to a multitude of intracellular biological states.

Here, we firstly identified the prognostic and oncogenic function of CPT1C in ESCC. We observed that CPT1C could promote G1/S transition, fatty acid utilization and tumor cell proliferation in ESCC. Moreover, increasing CPT1C protein status was associated with poor prognosis in clinical ESCC patients. Besides its role in facilitating tumor cell proliferation and survival, CPT1C has also been reported to play a supporting role in promoting gastric cancer ovarian metastasis [63], hepatocellular carcinoma metastasis [30] and colorectal cancer cell metastasis [31]. However, our in vitro data did not show a significant increase in cell motility when CPT1C was overexpressed, suggesting that the heterogeneity of different tumor types and subtypes is involved. Accordingly, more comprehensive and systematic studies are needed to elucidate the role of CPT1C in different types of tumors.

## Conclusions

**Fig. 8:**
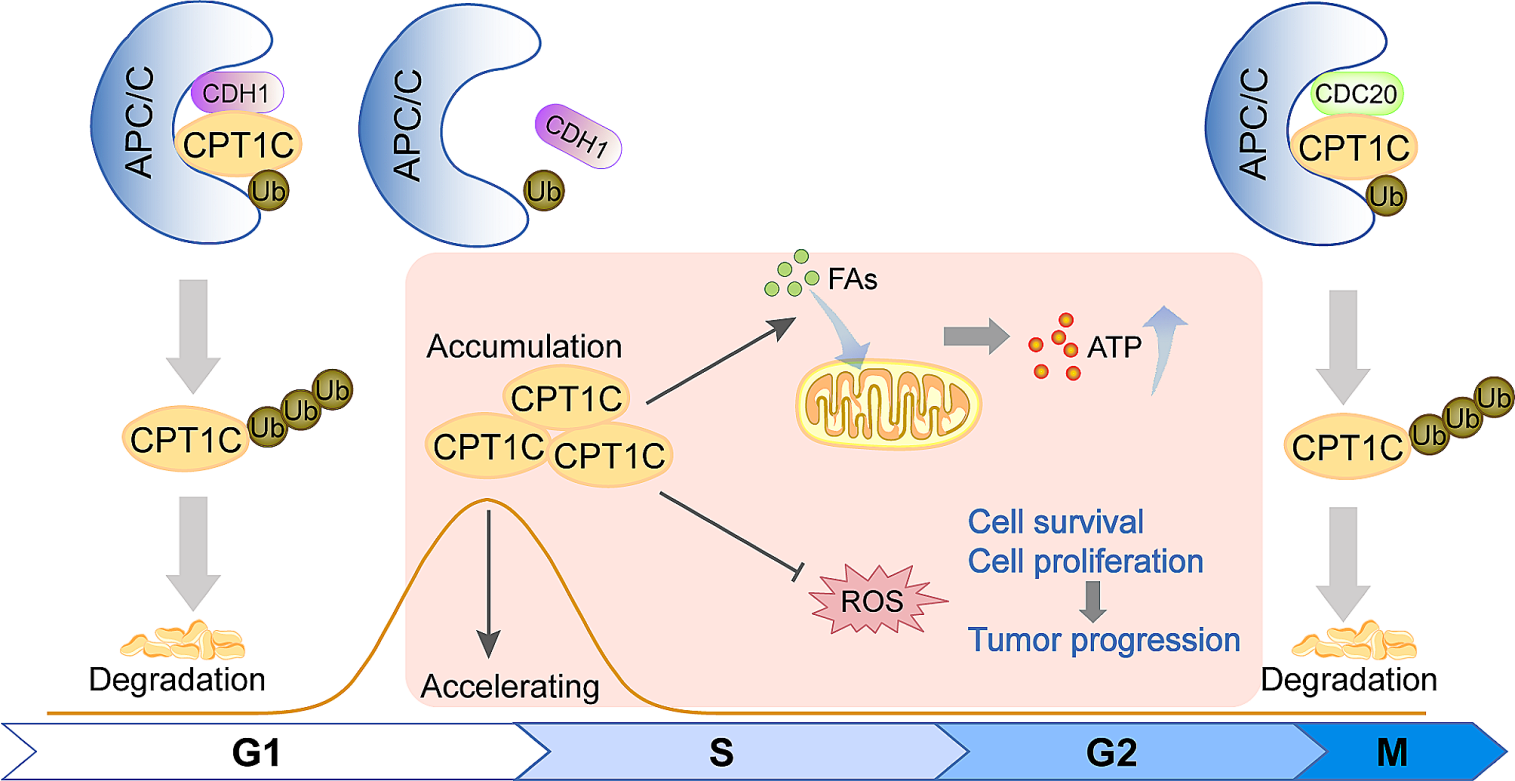
Schematic diagram illustrating the mechanism of APC/C-regulated CPT1C promotes tumor cells survival and proliferation

Cumulatively, our findings revealed a previously unknown connection between cell cycle progression and fatty acid metabolism. Additionally, CPT1C promotes tumor cell proliferation and survival by upregulating ATP levels and decreasing ROS levels, particularly under metabolic stress (Fig. 8). CPT1C likely plays a crucial role in ESCC progression and is a potential prognostic indicator for ESCC.

### Electronic supplementary material

Below is the link to the electronic supplementary material.

Supplementary Material 1

## Data Availability

No datasets were generated or analysed during the current study.

## Abbreviations

ACSL: Acyl-CoA synthetase long-chain
APC/C: Anaphase-promoting complex/cyclosome
ATP: Adenosine triphosphate
CDC20: Cell-division cycle protein 20
CDH1: CDC20 homolog 1
CDK: Cyclin-dependent kinase
CHX: Cycloheximide
CM: Control medium
CPT1: Carnitine palmitoyltransferase 1
DAPI: 4′,6-diamidino-2-phenylindole
DTB: Double-thymidine block
EdU: 5-ethynyl-2′-deoxyuridine
ESCC: Esophageal squamous cell carcinoma
FAO: Fatty acid oxidation
FBS: Fetal bovine serum
GLS1: Glutaminase 1
IHC: Immunohistochemistry
IP: Immunoprecipitation
LG: Low glucose
MTS: 3-(4,5-dimethylthiazol-2-yl)-5-(3-carboxymethoxyphenyl)-2-(4-sulfophenyl)-2 H-tetrazolium, inner salt
Noc: Nocodazole
OCR: Oxygen-consumption rate
OS: Overall survival
PFKFB3: 6-phosphofructo-2-kinase/fructose-2,6-biphosphatase 3
PI: Propidium iodide, PLA, proximity-ligation assay
ROS: Reactive oxygen species
SEM: Standard error of the mean
TCA: Tricarboxylic acid
