# Supplementary material for: APC/C-regulated CPT1C promotes tumor progression by upregulating the energy supply and accelerating the G1/S transition
Source: Cell Commun Signal. 2024 May 23;22:283. doi: 10.1186/s12964-024-01657-z (PMC11112774; doi:10.1186/s12964-024-01657-z)
Supplement: Supplementary file 1 — Supplementary Material 1 [file 12964_2024_1657_MOESM1_ESM.docx]

**Supplementary figure**


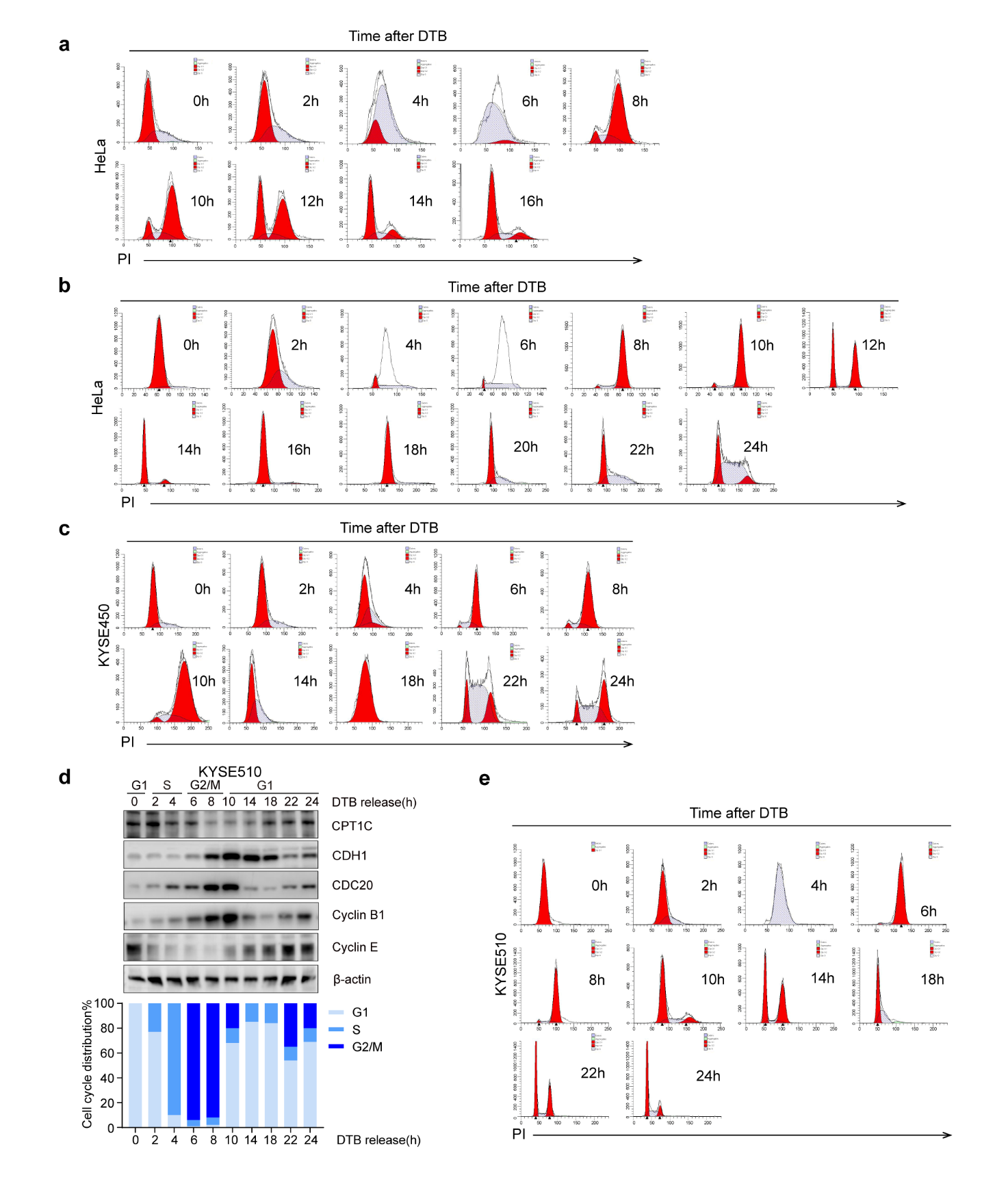


**Figure S1** **Expression of CPT1C appears in a cell cycle-dependent manner and gets peak in G1/S boundary**

**a** The cell cycle analysis of HeLa cells basing on PI-staining after double thymidine block treatment at indicated time points. **b** The cell cycle analysis of HeLa cells after double thymidine block treatment at indicated time points. **c** The cell cycle analysis of KYSE450 cells after double thymidine block treatment at indicated time points. **d, e** KYSE510 cells were arrested at the G1/S boundary after double thymidine block treatment, released into fresh medium, and harvested at the indicated times for immunoblotting (the upper panel of d) and flow cytometry analysis of cell cycle distribution (the down panel of d, and e). Three independent experiments were performed. DTB: double thymidine block.


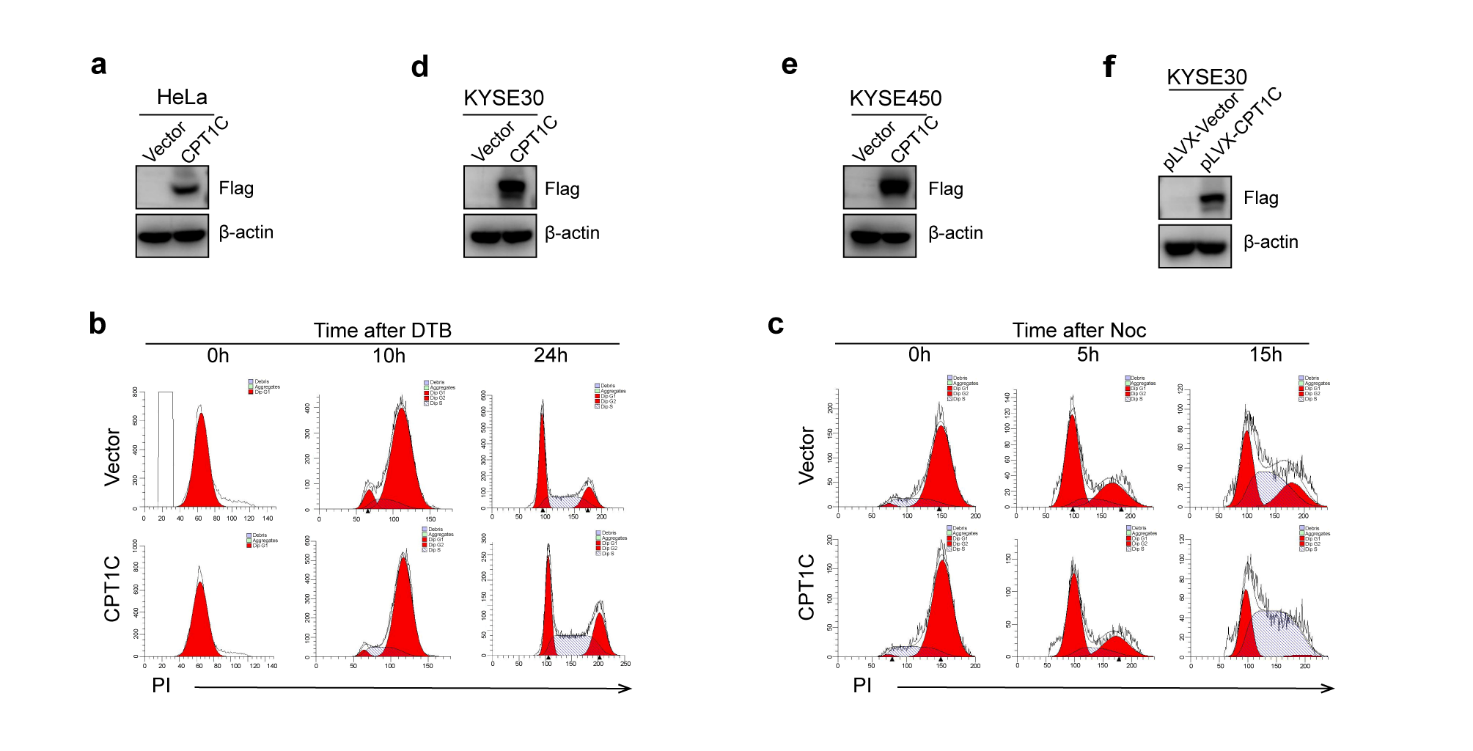


**Figure S2** **CPT1C accelerates G1/S transition and promotes malignant proliferation of tumor cells**

**a** Immunoblotting analysis of HeLa cells transfected with empty vectors and CPT1C plasmids. **b** The cell cycle analysis of HeLa cells after double thymidine block treatment for indicate duration. **c** The cell cycle analysis of HeLa cells after nocodazole treatment for indicate duration. **d** Immunoblotting analysis of KYSE30 cells transfected with empty vectors and CPT1C plasmids. **e** Immunoblotting analysis of KYSE450 cells transfected with empty vectors and CPT1C plasmids. **f** Immunoblotting analysis(right) of KYSE30 cells stably transfected with empty vectors and CPT1C plasmids. Three independent experiments were performed. DTB: double thymidine block，Noc: nocodazole.

| **Supplementary Table S1. Antibodies used in this study.** | | |
| --- | --- | --- |
| Name | Vendor | Catalog No. |
| CPT1C | Proteintech | 66072-1-Ig |
| CPT1A | Proteintech | 15184-1-AP |
| CPT1B | Proteintech | 22170-1-AP |
| CPT2 | Proteintech | 26555-1-AP |
| ACSL1 | Proteintech | 13989-1-AP |
| ACSL4 | Abcam | ab155282 |
| cyclin A | Santa Cruz | sc-53229 |
| cyclin B1 | CST | 12231 |
| cyclin E | Santa Cruz | sc-247 |
| CDH1 | Sigma | C7855 |
| CDC20 | CST | 14866 |
| CDC27(APC3) | Proteintech | 10918-1-AP |
| APC11 | CST | 14090 |
| APC2 | CST | 12301 |
| Ubiquitin | CST | 3933 |
| Flag (DYKDDDDK) Tag | Proteintech | 66008-4-Ig |
| Ki-67 | Abcam | ab15580 |
| VDAC1 | Proteintech | 55259-1-AP |
| ATP5A | Proteintech | 14676-1-AP |
| GAPDH | Proteintech | 60004-1-Ig |
| β-actin | Proteintech | 66009-1-Ig |
|  |  |  |

| **Supplementary Table S2. Associations between the expression of CPT1C and the clinicopathological characteristics.** | | | | | |
| --- | --- | --- | --- | --- | --- |
| Variables | CPT1C expression | | Total  (n) | *P* value | r value |
|  | Low(n) | High(n) |  |  |  |
| Gender |  |  |  | 0.11 | 0.175 |
| Male | 12 | 5 | 17 |  |  |
| Female | 40 | 45 | 85 |  |  |
| Age |  |  |  | 1 | -0.02 |
| ≤67.5 | 26 | 25 | 51 |  |  |
| >67.5 | 27 | 24 | 51 |  |  |
| Tumor size |  |  |  | 1 | **0.019** |
| ≤4cm | 27 | 26 | 53 |  |  |
| >4cm | 24 | 25 | 49 |  |  |
| T |  |  |  | 1 | **0.022** |
| Ⅰ-Ⅱ | 7 | 6 | 13 |  |  |
| Ⅲ-Ⅳ | 45 | 44 | 89 |  |  |
| N |  |  |  | 0.153 | 0.163 |
| N0 | 25 | 16 | 41 |  |  |
| /N1/N2/N3 | 27 | 34 | 61 |  |  |
| M |  |  |  | 0.114 | 0.178 |
| M0 | 52 | 47 | 99 |  |  |
| M1 | 0 | 3 | 3 |  |  |
| Grade |  |  |  | 0.377 | 0.1 |
| Ⅰ-Ⅱ | 40 | 34 | 74 |  |  |
| Ⅲ | 12 | 16 | 28 |  |  |
| TNM |  |  |  | 0.218 | 0.144 |
| Ⅰ-Ⅱ | 24 | 16 | 40 |  |  |
| Ⅲ-Ⅳ | 28 | 34 | 62 |  |  |

| **Supplementary Table S3. Cell cycle distribution in HeLa cells after** **double thymidine block.** | | | | | | | |
| --- | --- | --- | --- | --- | --- | --- | --- |
| DTB | | Vector (%) | | | CPT1C (%) | | |
| 0h | G1 | 100.00 | 100.00 | 100.00 | 100.00 | 100.00 | 100.00 |
|  | S | 0.00 | 0.00 | 0.00 | 0.00 | 0.00 | 0.00 |
|  | G2/M | 0.00 | 0.00 | 0.00 | 0.00 | 0.00 | 0.00 |
| 5h | G1 | 7.47 | 7.83 | 7.81 | 3.33 | 3.41 | 2.78 |
|  | S | 11.26 | 10.70 | 9.02 | 12.79 | 13.24 | 15.01 |
|  | G2/M | 81.28 | 81.48 | 83.17 | 83.88 | 83.35 | 82.21 |
| 15h | G1 | 45.74 | 46.23 | 46.30 | 31.12 | 32.28 | 32.13 |
|  | S | 33.90 | 33.17 | 33.21 | 42.52 | 41.04 | 41.41 |
|  | G2/M | 20.36 | 20.60 | 20.49 | 26.36 | 26.68 | 26.46 |

Note: DTB: double thymidine block.

| **Supplementary Table S4. Cell cycle distribution in HeLa cells after nocodazole block.** | | | | | | | |
| --- | --- | --- | --- | --- | --- | --- | --- |
| Nocodazole block | | Vector (%) | | | CPT1C (%) | | |
| 0h | G1 | 1.66 | 0.80 | 2.30 | 1.10 | 0.99 | 1.31 |
|  | S | 17.13 | 19.89 | 18.21 | 18.66 | 19.6 | 18.52 |
|  | G2/M | 81.21 | 79.31 | 79.49 | 80.24 | 79.41 | 80.16 |
| 5h | G1 | 56.98 | 62.59 | 54.85 | 55.60 | 59.63 | 58.37 |
|  | S | 13.09 | 10.89 | 10.36 | 16.96 | 9.79 | 18.65 |
|  | G2/M | 29.92 | 36.52 | 24.79 | 27.44 | 34.59 | 26.98 |
| 15h | G1 | 33.84 | 37.67 | 38.16 | 20.77 | 25.85 | 0.57 |
|  | S | 44.79 | 40.74 | 29.00 | 78.58 | 72.46 | 94.21 |
|  | G2/M | 21.38 | 21.59 | 32.84 | 0.65 | 1.70 | 5.23 |

| **Supplementary Table S5. The expression pattern of CPT1C protein in ESCC and paracancerous tissues.** | | | |
| --- | --- | --- | --- |
| CPT1C expression | ESCC tissue (n) | paracancerous tissue (n) | *P* value |
| High | 45 | 4 | <0.0001 |
| Low | 57 | 67 |  |

Note: ESCC: esophageal squamous cell carcinoma.

| **Supplementary Table S6. Univariate and multivariable Cox regression analysis of**  **overall survival (n=102).** | | | | |
| --- | --- | --- | --- | --- |
| Variables | Univariate analysis | | Multivariate analysis | |
|  | HR (95%CI) | *P* value | HR (95%CI) | *P* value |
| CPT1C expression | 1.996(1.23-3.329) | **0.00516** | 1.71(1.03-2.84) | **0.0394** |
| Gender | 1.733(0.858-3.501) | 0.125 |  |  |
| Age | 0.767(0.474-1.24) | 0.279 |  |  |
| Tumor_size | 1.695(1.047-2.745) | **0.0318** | 1.97(1.19-3.25) | **0.00837** |
| T | 1.623(0.742-3.549) | 0.225 |  |  |
| N | 2.025(1.206-3.401) | **0.00765** | 0.91(0.35-2.34) | 0.837 |
| M | 3.601(1.114-11.637) | **0.0323** | 1.08(0.25-4.63) | 0.917 |
| Grade | 0.993(0.585-1.684) | 0.978 |  |  |
| TNM | 2.443(1.43-4.175) | **0.00108** | 2.89(1.09-7.69) | **0.0333** |

Note: HR: hazard ratio; 95%CI: 95% confidence interval.
